# Supplementary material for: ID2 Promotes Lineage Transition of Prostate Cancer through FGFR and JAK-STAT Signaling
Source: Cancers (Basel). 2024 Jan 17;16(2):392. doi: 10.3390/cancers16020392 (PMC10814654; doi:10.3390/cancers16020392)
Supplement: Supplementary file 1 [file cancers-16-00392-s001.zip › Supplementary Figure.pdf]

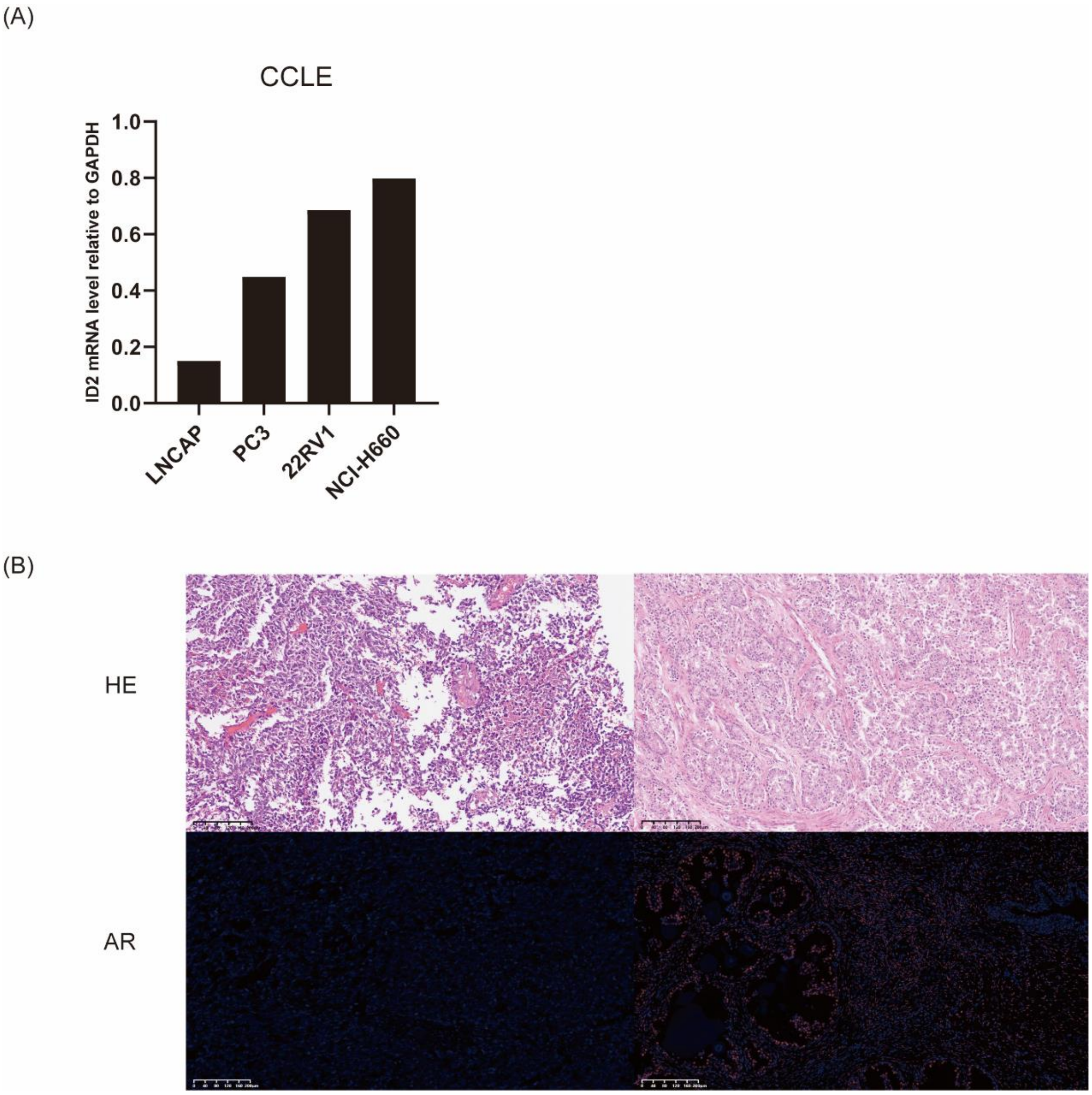

Figure S1. ID2 Expression Is Upregulated in NEPC and DNPC. (**A**) Relative ID2 expression level in PCa cell lines from CCLE. (**B**) HE staining and immunofluorescent of clinical PCa patients.

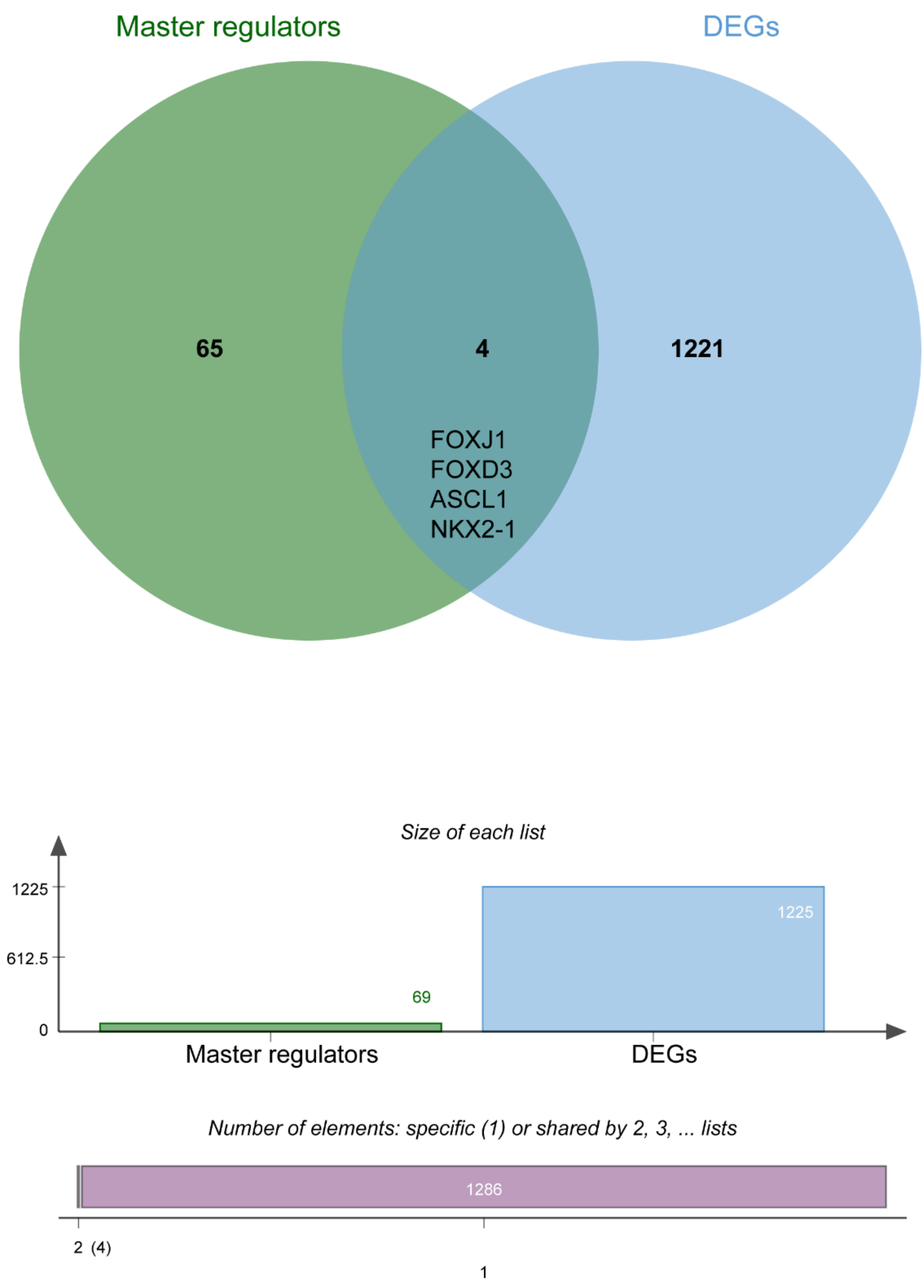

Figure S2. Transcriptomic Reprogramming and Pathway Analysis upon ID2 Overexpression. Intersection of Master regulators and DEGs. Master regulators was identified by viper R packages to infer effects of ID2 on protein activity changes. DEGs was identified using RNA-seq data of clinical NEPC and ADPC samples from SU2C 2019.

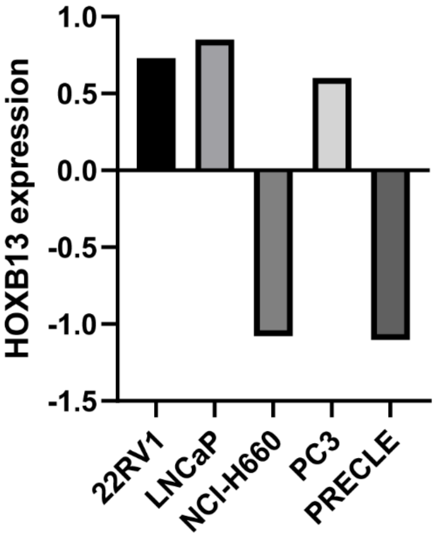

Figure S3. ID2 Attenuates AR Signaling and Promotes NETD. Z-score normalized HOXB13 mRNA expression in PCa cell lines from CCLE.

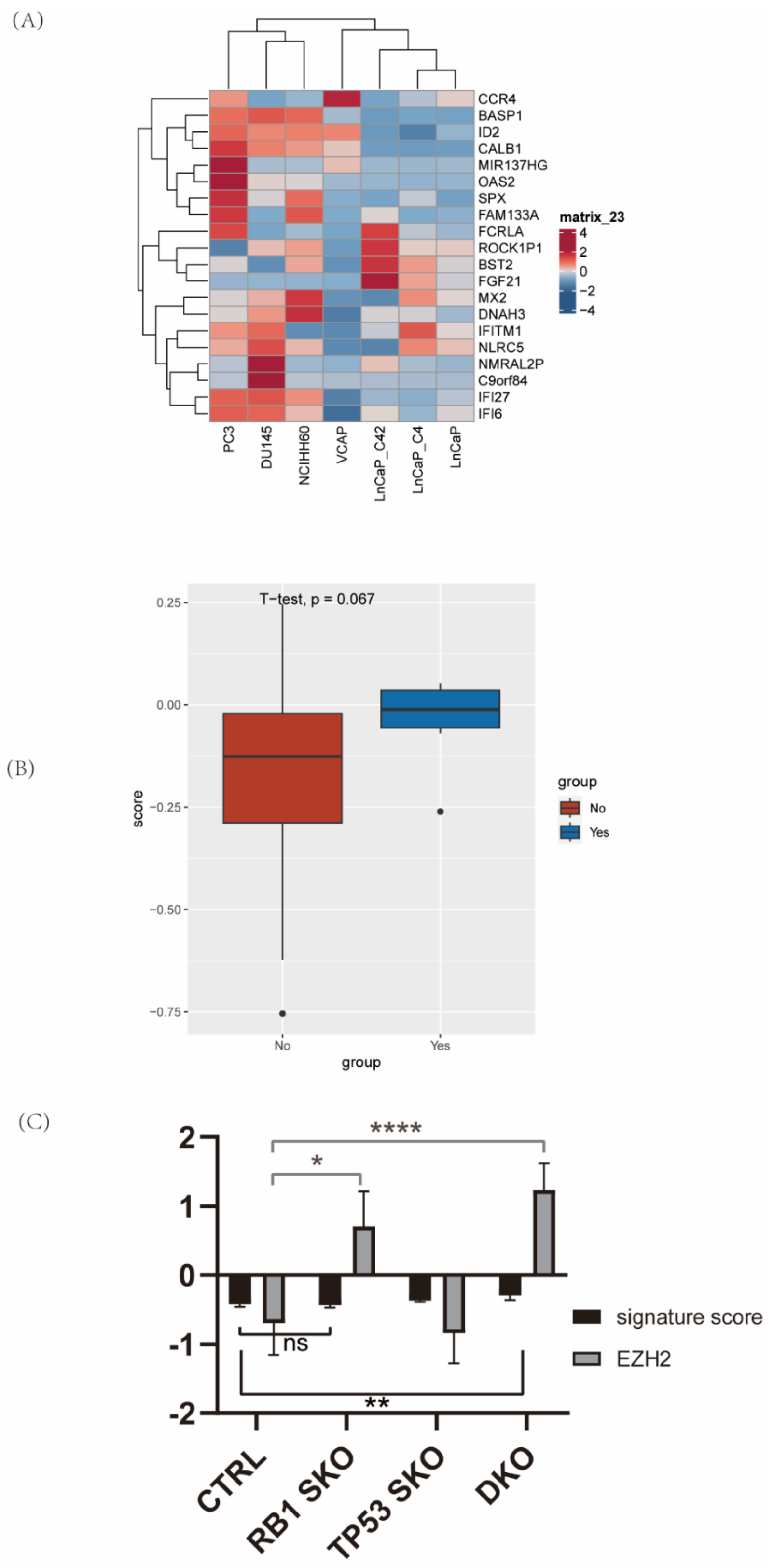

Figure S4. ID2 UP50 Signature Generation and Validation of Its Effectiveness. (**A**) Expression of top 20 DEGs in PCa cell lines. (**B**) ID2 UP50 signature score in clinical sample with NE features or not. (**C**) ID2 UP50 signature analysis and EZH2 expression analysis after RB1 or TP53 gene knock out in LNCaP cell. SKO: single knock out. DKO: double knock out.

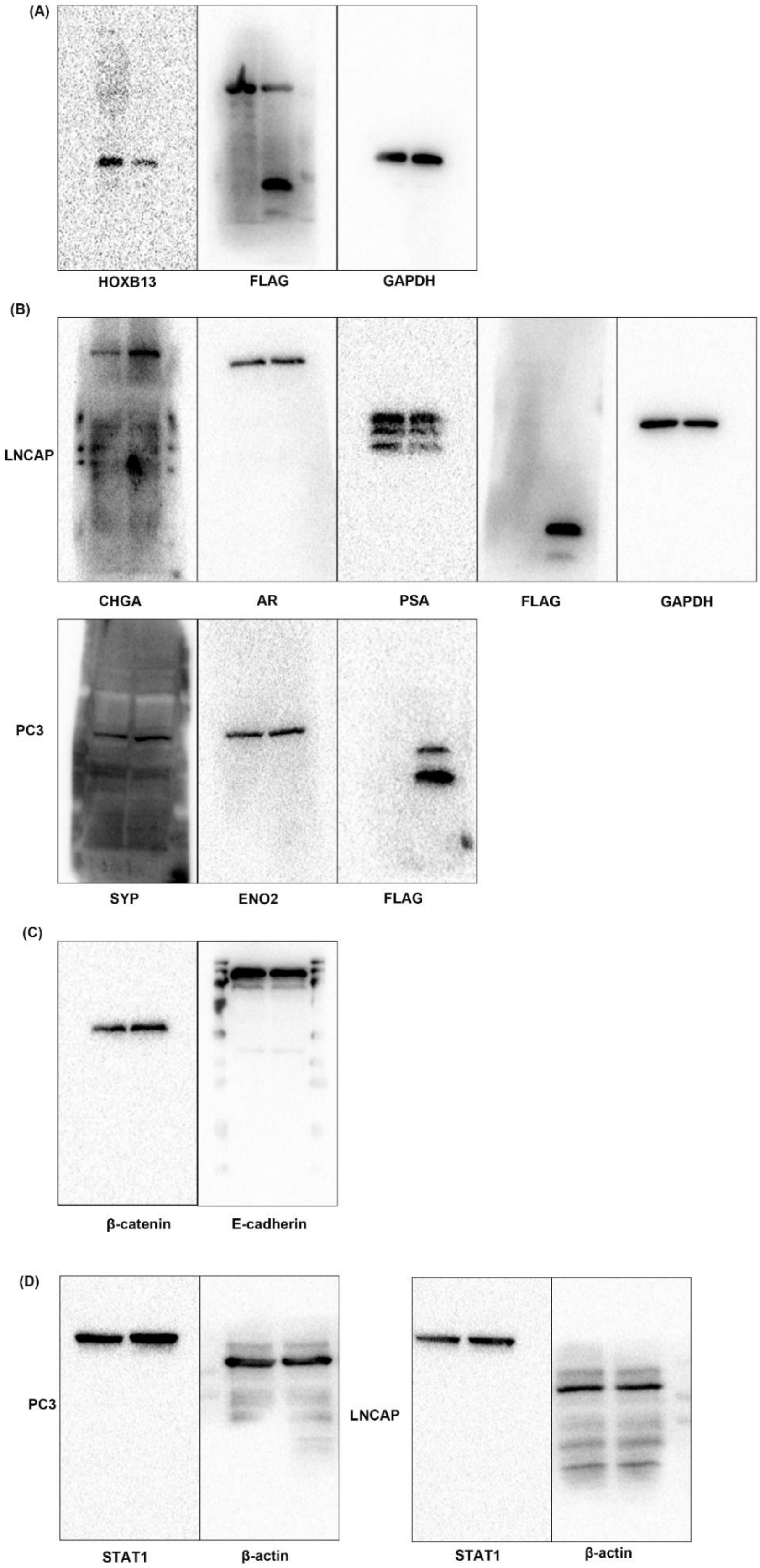

Figure S5. Original blot of this study. (**A**) Original blot of Figure 2E. (**B**) Original blot of Figure 3A. (**C**) Original blot of Figure 4G. (**D**) Original blot of Figure 6E.
